# Location of the Proposed State Insane Asylum

**Published:** 1869-10

**Authors:** 


### Location of the proposed State Insane Asylum.

We have no doubt all our readers, and all intelligent fair-minded men, will at once see the fitness of the location selected by the Commissioners who were appointed by the Governor for this purpose. We understand that Buffalo was the unanimous recommendation of the commission. This is creditable in the highest degree; the more so when we remember that the men who composed the commission were representatives of towns all anxious for the new institution, and all liberal in offering suitable sites for this purpose. A little reflection will satisfy every one that Buffalo is really the only place which could be recommended to the legislature with any unanimity, though all the towns offering sites are in themselves beautiful, attractive and healthy.

The location of such an institution is a matter of great importance; and if the site had been selected upon any other than the most liberal and high-minded principles, it would prove to Western New York and vicinity an irretrievable loss. The recommendation of the commission shows the wisdom of the Governor in his selection of men. They were all alive to the claims of their respective places, and doubtless see reasons why their own towns should receive the location above any other, but still they were not blinded by prejudice—they were not demoralized by party—they had no political or personal ends to gain—they were honest, fair-minded, intelligent citizens, and were anxious only to promote the general good. If many more of our public interests could be committed to such guardianship, better for the State and vastly better for the people; but, alas! politicians and men who desire and hold office are made of different stuff.

The wise liberality of the Common Council of the city of Buffalo is also worthy of notice. With great unanimity of purpose, and without any of the usual divisions and oppositions, the commission were invited to select a suitable place, at the expense of the city, not exceeding $50.000, the city to furnish the same with ample supply of water perpetually. This was all that could be asked, and it was cheerfully and unanimously granted.

It is believed that the recommendation of the commission will meet the hearty approval of all interested, and that the next legislature will show the same liberality in furthering the object that all parties thus far engaged in the enterprise have manifested.

